# Supplementary material for: Current Situation of Bacterial Infections and Antimicrobial Resistance Profiles in Pet Rabbits in Spain
Source: Vet Sci. 2023 May 14;10(5):352. doi: 10.3390/vetsci10050352 (PMC10221308; doi:10.3390/vetsci10050352)
Supplement: Supplementary file 1 [file vetsci-10-00352-s001.zip › vetsci-2359827-supplementary.pdf]

Supplementary Table S1. Minimal Inhibitory Concentrations (MIC) in different bacterial isolates and antimicrobials.

	Gram-negative												Gram-positive			
	Acinetobacter spp		*Pseudomonas* spp		*Bordetella* spp		*Escherichia* spp		*Klebsiella* spp		*Enterobacter* spp		*Staphylococcus* spp		*Enterococcus* spp	
Ab	MIC_{50}	MIC_{90}	MIC_{50}	MIC_{90}	MIC_{50}	MIC_{90}	MIC_{50}	MIC_{90}	MIC_{50}	MIC_{90}	MIC_{50}	MIC_{90}	MIC_{50}	MIC_{90}	MIC_{50}	MIC_{90}
AMP	16	16	16	32	16	16	16	32	16	32	16	24	4	8	4	4
AMC	8	16	16	32	8	8	8	16	8	16	16	32	4	8	4	4
LEX	1	4	1	8	1	4	1	1	1	8	1	4	4	8	4	8
CTX	8	16	16	32	32	64	1	16	4	32	1	32	1	32	2	16
IMP	1	4	1	8	1	4	1	1	1	8	1	4	4	8	4	8
GEN	2	8	4	8	4	8	2	8	6	8	2	8	1	8	8	8
AMK	8	8	8	32	16	16	8	16	8	32	8	16	8	32	32	32
CHL	8	16	16	32	8	27	8	16	8	16	8	16	8	8	8	15.2
FOS	32	64	32	64	64	64	32	32	32	32	32	64	32	32	32	32
COL	1	11.6	1	8	1	4	1	7.2	1	8.8	1	23	NA	NA	NA	NA
VAN	NA	NA	NA	NA	NA	NA	NA	NA	NA	NA	NA	NA	1	2	2	2.4

AMP, ampicillin; AMC, amoxicillin clavulanic acid; LEX, cephalexin; CTX, cefotaxime; IMP, imipenem; GEN, gentamycin; AMK, amikacin; CHL, chloramphenicol; FOS, fosfomycin; COL, colistin; VAN, vancomycin.

NA, not applicable
